# Functional Connectivity between Face-Movement and Speech-Intelligibility Areas during Auditory-Only Speech Perception

**DOI:** 10.1371/journal.pone.0086325

**Published:** 2014-01-23

**Authors:** Sonja Schall, Katharina von Kriegstein

**Affiliations:** 1 Max Planck Institute for Human Cognitive and Brain Sciences, Leipzig, Germany; 2 Humboldt University of Berlin, Berlin, Germany; Harvard Medical School/Massachusetts General Hospital, United States of America

## Abstract

It has been proposed that internal simulation of the talking face of visually-known speakers facilitates auditory speech recognition. One prediction of this view is that brain areas involved in auditory-only speech comprehension interact with visual face-movement sensitive areas, even under auditory-only listening conditions. Here, we test this hypothesis using connectivity analyses of functional magnetic resonance imaging (fMRI) data. Participants (17 normal participants, 17 developmental prosopagnosics) first learned six speakers via brief voice-face or voice-occupation training (<2 min/speaker). This was followed by an auditory-only speech recognition task and a control task (voice recognition) involving the learned speakers’ voices in the MRI scanner. As hypothesized, we found that, during speech recognition, familiarity with the speaker’s face increased the functional connectivity between the face-movement sensitive posterior superior temporal sulcus (STS) and an anterior STS region that supports auditory speech intelligibility. There was no difference between normal participants and prosopagnosics. This was expected because previous findings have shown that both groups use the face-movement sensitive STS to optimize auditory-only speech comprehension. Overall, the present findings indicate that learned visual information is integrated into the analysis of auditory-only speech and that this integration results from the interaction of task-relevant face-movement and auditory speech-sensitive areas.

## Introduction

Even though speech is primarily conveyed acoustically, speech comprehension is influenced by the visible facial kinematics of the speaker. Seeing the moving mouth, lips and tongue of a speaker improves speech comprehension substantially [1]–[3]. Visual speech cues are particularly powerful under noisy conditions [1]–[3], and in cases when the listener is familiar with a speaker’s face and voice [4].

Familiarity with a speaker’s face can affect speech comprehension even under auditory-only listening conditions; a brief familiarization with a speaker’s visual or audiovisual speaking dynamics increases the subsequent recognition performance in auditory-only speech recognition tasks [5], [6]. In addition, another aspect of auditory communication, i.e., the recognition of the speaker’s identity, is also improved [5], [7], [8]. On the neural level, these behavioral benefits are associated with the activation of face-sensitive brain areas, i.e., the face-movement sensitive posterior STS (pSTS), which is associated with recognition of what is said (speech recognition), and the face-identity sensitive fusiform face area (FFA), which is associated with recognition of who is speaking (voice recognition).

Based on these findings, it has been suggested that the brain uses a simulation of the speaker’s talking face to optimize auditory perception, even in auditory-only conditions [5]. This idea is part of an audiovisual model of communication that has been explicitly described in [5], [9]. One of the core features of this model is that specific visual, face-sensitive areas are also activated under auditory-only conditions thereby helping the auditory system to recognize a speaker’s identity and speech. This means that areas sensitive to face-identity support auditory-only voice recognition and areas sensitive to visual speech movement support auditory-only speech recognition. An integration mechanism that allows information in face-sensitive cortices to be shared with voice−/speech- processing regions under auditory-only conditions is an important pre-requisite for the audiovisual model of communication.

**Figure 1:**
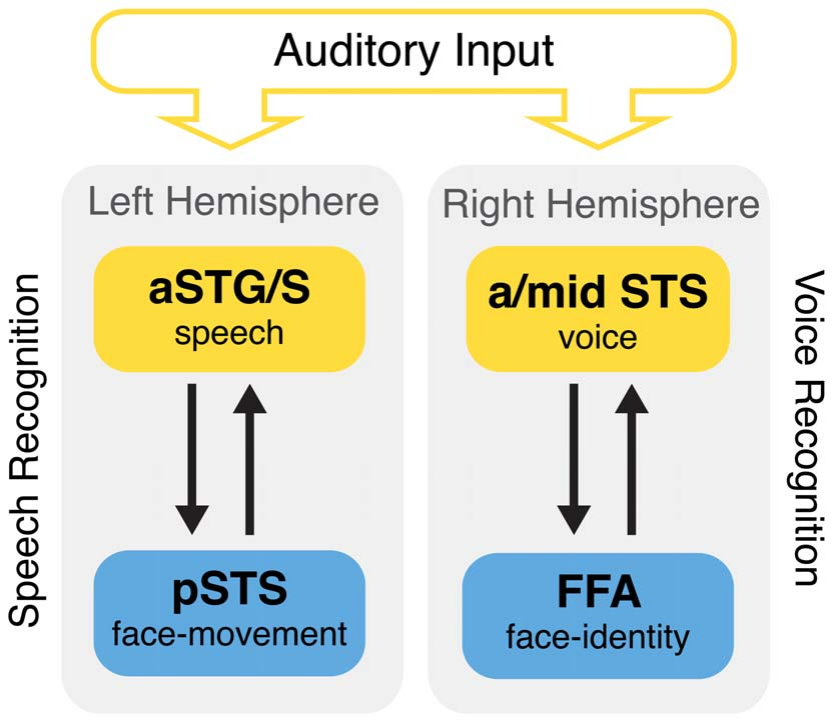
Hypotheses. Visual information about a speaker is integrated into the auditory analysis via functional connections (black arrows) between face-sensitive regions (blue) and higher-level auditory regions (yellow). Previous studies have shown that, during voice recognition, functional connectivity increases between the face-identity sensitive FFA and the voice-sensitive anterior/mid STS in the right temporal lobe (right side of the figure) (von Kriegstein and Giraud, 2006). In the present study, we tested whether, during speech recognition, functional interactions exist between the face-movement sensitive pSTS and speech-intelligibility sensitive areas in the aSTG/S in the left temporal lobe. In addition, we tested whether the integration of visual information into auditory information relies on the ability to recognize faces, by comparing a group of developmental prosopagnosics (i.e., people with a face-recognition deficit) with a group of normal participants. aSTG/S: anterior superior temporal gyrus/sulcus.

Studies investigating simultaneous input of auditory and visual speech information have revealed interactions between visual areas and low-level auditory sensory areas [10], [11]. However, functional connections might be fundamentally different when only auditory input is available, compared to when both, voice and face input is present. For example, studies investigating auditory-only voice recognition suggest that auditory and visual speaker information is shared by direct functional and structural connections between relatively high-level sensory areas, i.e., the FFA, and anterior/middle voice-sensitive areas in the temporal lobe (Fig. 1, right; [8], [12], [13]). In how far, this mechanism translates to auditory-only speech recognition is currently unknown. Here, we address this question by investigating the functional connectivity of the face-movement sensitive pSTS during auditory-only speech recognition of visually-known speakers using functional magnetic resonance imaging (fMRI) data. Based on the audiovisual model of communication, we hypothesized an integration mechanism between the face-movement sensitive pSTS and an auditory higher-level speech area during speech recognition (Fig. 1, left).

**Figure 2:**
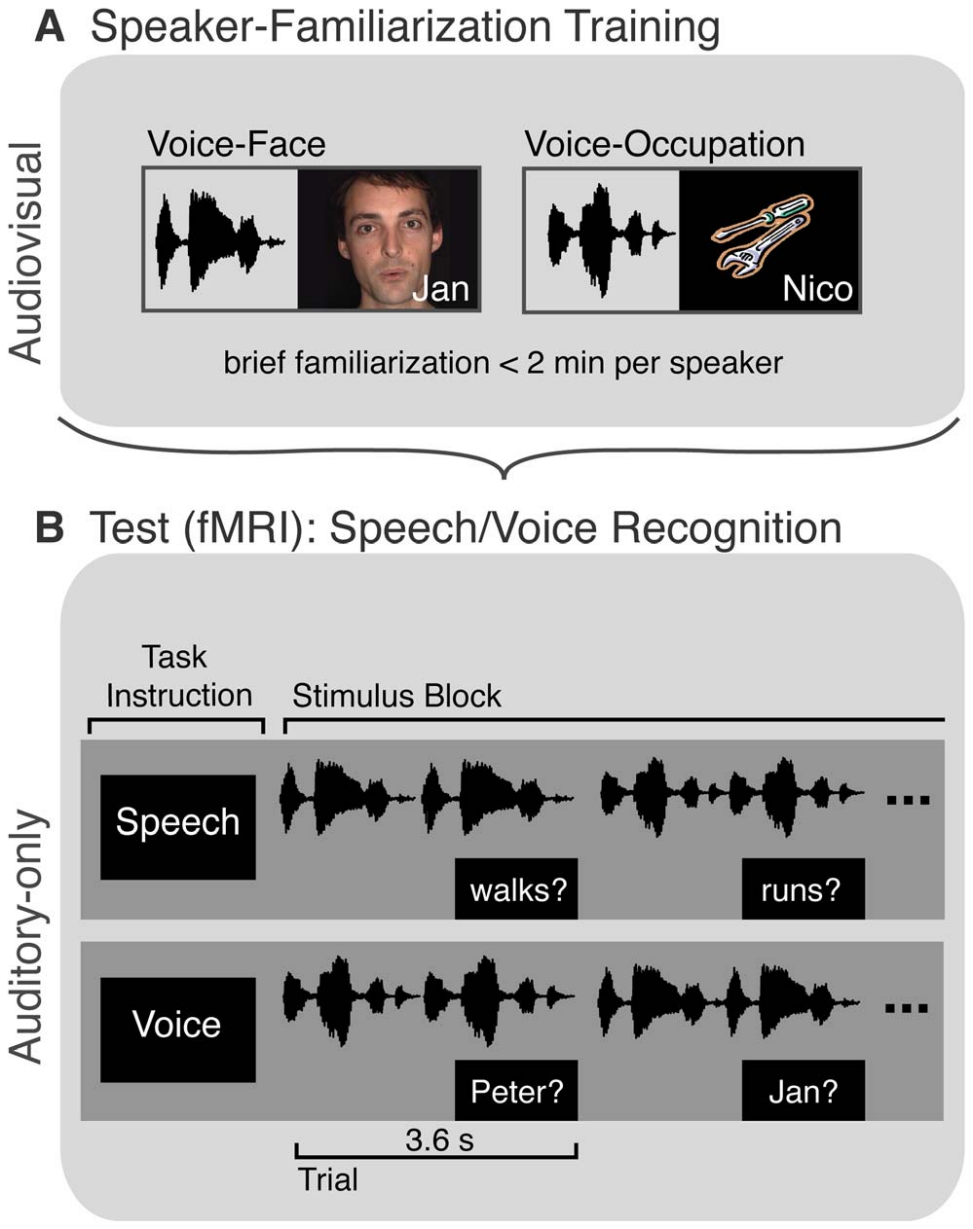
Experimental Paradigm. (**A**) Prior to fMRI-scanning, participants received audiovisual training to familiarize them with six male speakers. Three speakers were learned by voice, name, and a video showing the speaker’s talking face (voice-face learning), while the other three speakers were learned by voice, name, and a picture illustrating an occupation (voice-occupation learning). (**B**) FMRI data were acquired in the subsequent auditory-only test-phase. Participants listened to sentences recorded by the six speakers and performed either a speech recognition task (“Did that word occur in the sentence?”) or voice recognition task (“Does the name match the voice identity?”). A written word (speech task) or name (voice task) came on during the last second of the auditory stimulus presentation to prompt the participant to response. Task instructions were given at the beginning of each stimulus block.

While auditory speech is, in general, processed by a wide network of brain regions, recent neuroimaging and brain stimulation studies suggest that the left anterior superior temporal gyrus and sulcus (aSTG/S) play a particular role in processing intelligible speech and are crucial for successful comprehension of auditory speech [14]–[20]. We therefore considered the aSTG/S may be a prime candidate to be the brain area that is functionally connected to the face-sensitive pSTS during auditory-only speech recognition. To test for an integration mechanism between aSTG/S and pSTS, we used data from a previous fMRI experiment that investigated speech recognition after brief audio-visual, voice-face training in contrast to a matched control training (voice-occupation training). The design additionally included a voice-recognition task on the same stimulus material (Fig. 2) [5]. This data set was acquired from a subject group with normal face perception abilities and a subject group of developmental prosopagnosics, i.e., people who have a face-identity recognition deficit [21], [22]. In addition to addressing our main research question, this allowed us to test whether the connectivity between auditory brain areas and face-sensitive brain areas depends on the ability to recognize faces. A previous case report suggested that this is not the case, at least for the connectivity between voice-sensitive and face-sensitive areas (Fig. 1, left). A replication of this finding for a group of prosopagnosics as well as its extension to speech-intelligibility and face-movement areas (Fig. 1, right) would indicate that a functional connection between face-sensitive and auditory regions is established at sensory processing stages rather than at stages of person identification.

## Methods

In the study, all participants were first trained on a set of voices together with a corresponding talking face (voice-face training), and on another set of voices with a symbol of the occupation of the speaker (voice-occupation training). This audio-visual training was followed by an auditory test phase in the MRI scanner. During the auditory test phase, speech recognition and voice recognition performance was tested using auditory-only speech samples (Fig. 2). After that, a visual face area localizer was acquired. In the following, we will detail the methods including participants, stimuli, and experimental design.

### Participants

In total, 34 right-handed volunteers participated in the study. 17 volunteers were normal participants (mean age: 37.4 years; 10 female) and 17 matched developmental prosopagnosics (mean age: 37.2 years; 11 female).

### Ethics Statement

The study has been approved by the Frankfurt Medical University as the responsible local ethics committee. All participants gave their written informed consent in accordance with the local ethics committee requirements.

### Stimuli

For the audio-visual training, stimulus material consisted of video and sound recordings of 6 male actors. Twenty second-person, interrogative, neutral German sentences (e.g., “Hast du dich beeilt?” *Did you hurry?*) were used. For the auditory test phase, stimulus material consisted of sound-only recordings of the same six male actors. The stimuli included 20 sentences in third-person, declarative form (e.g., “Er hat sich beeilt” *He hurried.*). The 40 sentences for the audio-visual training and auditory test were recorded from each speaker using a digital video camera (DCR-PC01E, Sony; 32-kHz sampling rate, 16-bit resolution). Auditory stimuli were equalized for overall sound pressure using CoolEdit (Syntrillium Software). Pictures of occupation symbols for the voice-occupation training condition were taken from http://office.microsoft.com/en-us/clipart. For the visual face area localizer, still frames of faces were taken from the recordings of 36 additional speakers. Visual objects were photographs of 36 different objects. All photographs were in full color and were 768×576 pixels in size.

### Procedure & Experimental Design

The experiment was a 2×2×2 factorial design with the within-subjects factors training-type (voice-face training, voice-occupation-training) and task (speech, voice) and the between-subjects factor group (normal participants, developmental prosopagnosics). All participants performed the audio-visual training first, and then the auditory test (Fig. 2).

### Audio-visual Training Phase

Before scanning, participants completed an audiovisual training phase that familiarized them with the voices of six male speakers (Fig. 2A). Voices were learned in two different conditions: voice-face training and voice-occupation training. For voice-face training, participants watched video-clips of three talking speakers, i.e., voices were learned in association with the speaker’s moving face. In the voice-occupation training condition, participants listened to the same sentences spoken by three other speakers, in association with a static symbol that visualized the speaker’s occupation. This audio-visual control condition ensured that all voices were learned together with a visual stimulus that contained person-related information. Although voice-face and voice-occupation training conditions differed regarding their dynamic structure, previous analyses have shown that the difference in movement-perception between the two conditions cannot explain the crossmodal activation of face-sensitive regions and the associated behavioral benefit (see discussion in von Kriegstein et al., 2008). In both training conditions, participants also learned the speaker’s name. Each participant learned three of the six voices with a face and three with an occupation. Face- and occupation learned voices were counterbalanced across participants. The training consisted of learning-trials. A learning trial started with the presentation of a speaker’s name (1 s) and was either followed by a video-clip (face-voice training) or a voice recording together with an occupation symbol (voice-occupation training) (∼1.3 s). After a cycle of training (20 voice-face trials or 20 voice-occupation trials per speaker), learning was evaluated (8 trials per speaker). In the evaluation-trials, voices were followed by a name (4 trials/speaker) or face/occupation (4 trials/speaker) and participants had to indicate by button press whether the two sequentially presented stimuli matched in identity. Participants received feedback on the correctness of their choice. The learning-evaluation cycle was repeated at least twice for each condition (voice-face and voice-occupation learning). If participants did not reach the learning-criterion of at least 80% correct responses, the cycle was repeated a third time. All participants reached that criterion after 10 min training, i.e. a maximum of three learning cycles (<2 min per speaker).

### Auditory Test Phase

The test phase (Fig. 2B) took place during MRI-scanning. Stimuli were organized in a randomized block design with 12 blocks per condition. A block started with the presentation of a written word (either “speech” or “voice”) on the screen that informed the participant which task to perform. Within one block, there were eight trials of 3.6 s. A trial consisted of two sentences spoken by the same speaker. In the last second of each trial a written word/name was presented on the screen and participants had to indicate via button press whether the word had occurred in the preceding sentences (speech task) or whether the name matched the speaker’s identity (voice task). Crucially, participants performed the two tasks on the same auditory stimulus material. In addition, the design included a task with vehicle sounds, in which participants indicated via button press whether the sounds matched the written word (e.g., car, train). This condition was not included in the analysis. For details on these stimuli, see von Kriegstein et al. [5]. The MRI recordings were separated into 3 sessions each lasting 15 minutes. In between the sessions, participants were allowed to rest for a couple of minutes.

### Visual Face Area Localizer

To localize visual face-sensitive areas, participants passively watched 25 s blocks of colored photographs. Each photograph was presented for 0.5 s without any pause between stimuli. Blocks were separated by an interstimulus interval of 18 s during which participants saw a fixation cross. Photographs within one block showed either i) different faces with different facial gestures, ii) the same face with different facial gestures, iii) different objects from different view points, or iv) the same object from different view points. In condition i) the stream of pictures gave the impression of individual, static faces, while condition ii) gave the impression of a moving (talking) face of one identity. There were four blocks per condition. FMRI recordings were separated into two sessions of each 6 min.

### Data Acquisition

Structural and functional MRI was performed using a 3-T Siemens Vision scanner (gradient booster, standard head coil). Functional data were acquired using echo-planar imaging (whole brain coverage; 33 slices; 1 mm gap; voxel size: 3×3×3 mm; time to repeat (TR): 2 s). For the main experiment, 460 volumes were collected per session and participant. For the localizer, a total of 340 volumes were collected. Data can be made available upon request (Email: kriegstein@cbs.mpg.de).

### Data Analysis

#### Preprocessing and activity analysis

All data analyses were performed using SPM5 (www.fil.ion.uck.ac.uk/spm) and MarsBar (Brett et al, 2002) using standard procedures. For spatial preprocessing, scans were realigned, normalized to MNI space and smoothed using a Gaussian smoothing kernel of 8 mm full width at half maximum (FWHM) for group analyses and 4 mm FWHM for selection of seed regions in single participants. For activity analyses, statistical parametric maps were generated by modeling the evoked hemodynamic response for the different conditions as boxcars convolved with a synthetic hemodynamic response function in the context of the general linear model [23].

#### Psycho-physiological Interaction analysis (PPI)

Functional connectivity was assessed by psycho-physiological interactions (PPI) (Friston et al., 1997). For each participant, two separate analyses were performed to estimate 1) functional connectivity between areas sensitive to facial movement and auditory speech-intelligibility sensitive areas in left aSTG/S during speech recognition, and 2) functional connectivity between face- and voice-sensitive regions during voice recognition. Seed regions, covariates and regions of interest for the two different analyses are described in detail below.

Seed regions for **analysis 1** (face-movement sensitive pSTS) were the participant-specific parts of the pSTS that showed cross-modal activation in response to face-learned voices during a speech recognition task. The seed regions were identified in each individual by finding the maximum of the interaction contrast ((speech task/voice-face>speech task/voice-occupation)>(voice task/voice-face>voice task/voice-occupation)) that was located within a radius of 10 mm from the maximum of the face-movement sensitive pSTS in each individual subject (see Table S1). The face-movement sensitive pSTS was localized by the contrast, moving faces>static faces, of the visual face area localizer. Four participants did not have face-movement-sensitive voxels in the left pSTS. In these cases the group coordinate for the contrast, moving faces versus static faces, was used (random effects model, *p*<0.001 uncorrected). Four participants (2 normals, 2 prosopagnosics) who did not have cross-modal pSTS activation were excluded from this PPI analysis.

The seed regions for **analysis 2** (FFA) were those portions of the FFA that showed cross-modal activation in response to face-learned voices during the voice-recognition task. These seeds were identified in individual participants by finding the maximum of the interaction contrast ((voice task/voice-face>voice task/voice occupation)>(speech task/voice-face>speech task/voice-occupation)) that was located within a radius of 10 mm from the maximal FFA activity obtained by the visual face area localizer (see Table S2). Face-sensitive regions in the fusiform gyrus were localized in individual participants by the contrast, faces>objects. Four participants (2 normals, 2 prosopagnosics) who did not have cross-modal FFA activation were excluded from this analysis.

Covariates (first Eigenvariate from seed region, psychological variable, PPI regressor) were created using routines implemented in SPM5. The first eigenvariate was extracted from seed regions in each individual subject. The psychological variable was the speech task interaction contrast: ((speech task/voice-face>speech task/voice-occupation)>(voice task/voice-face>voice task/voice-occupation)) for **analysis 1** and the voice task interaction contrast: ((voice task/voice-face>voice task/voice-occupation)>(speech task/voice-face>speech task/voice-occupation)) for **analysis 2**. These interaction contrasts controlled for two things: (i) that connectivity is specifically increased for face-voice associations and not for associated visual stimuli in general, e.g. an occupation symbol, and (ii) that the increase in connectivity is specific for the task. This means that the speech task selectively induces increased functional connectivity between auditory speech areas and face-movment sensitive pSTS and the voice task selectively induces increased functional connectivity between auditory voice areas and face-identity sensitive FFA. For each analysis, the PPI regressor, the psychological variable, and the first Eigenvariate were entered as covariates in a design matrix at the single-subject level. Population-level inferences about BOLD (blood-oxygen dependent) signal changes were based on a random effects model that estimated the second-level statistic at each voxel using a one-sample *t*-test. Group level differences (developmental prosopagnosics vs normal participants) and the common analysis of both groups (developmental prosopagnosics and normal participants) were estimated at each voxel using a two-sample *t*-test.

Statistical evaluation was performed within regions of interest (ROI). For **analysis 1** the hypothesized target region was the speech-intelligibility sensitive aSTG/S in the left hemisphere (Fig. 1). There are several studies reporting this area in experiments investigating speech intelligibility [14]–[17], [24]. As a coordinate for the ROI, we first chose the one reported in a study by Friederici et al. [14], because that fMRI study was well controlled for voice related parameters like pitch. Thus, to determine the ROI we centered a sphere (r = 8 mm) on the peak coordinate (x,y,z = [−58,−4,4]). In a confirmatory analysis, we additionally tested a selection of aSTG/S coordinates taken from other fMRI and PET studies and a meta-analysis [15]–[17], [24]. The PPI results were considered significant if they were at a statistical threshold of *p*<0.05 FWE corrected for the ROI.

We also tested whether the functional connectivity might be mediated by heteromodal regions, because it has traditionally been assumed that interactions between modality specific regions occur exclusively via these heteromodal or multisensory regions [25]. However, over the last decade several studies have shown that there are direct structural and functional connections between auditory and visual modality-specific cortices, suggesting that audiovisual integration does not necessarily involve a multisensory cortical region [8], [12], [26]–[28]. To assess the possibility that a multisensory region mediates the functional connectivity between auditory and visual areas, we tested for increased functional connectivity to several multisensory regions: The pSTS–a region implicated in integrating audiovisual speech [18], [29]–[33], the inferior frontal gyrus (IFG) –which is thought to be involved in the perceptual mapping of visual speech and phonetic information [18], [34]–[36], and the inferior parietal lobule–which has been consistently found in studies investigating audiovisual speech integration [29], [35]. To build ROIs of these potential mediating areas, we took previously published coordinates from [18], [29]–[31], [35], [36]. Each coordinate served as a center of a spherical ROI (r = 8). Functional connectivity with the face-movement sensitive pSTS was statistically evaluated for each ROI independently.

For **analysis 2** the hypothesized target region was the right hemispheric voice-sensitive area (Fig. 1). This area was defined by the main effect, voice task>speech task, on the group level for normal participants (128 voxels, z = 2.82), developmental prosopagnosics (224 voxels, z = 3.58), and both groups together (2992 voxels, z = 4.24). The PPI results were considered significant if they were present at a statistical threshold of *p*<0.05 FWE corrected for the ROI.

## Results

In the following sections we will describe the results of the functional connectivity analyses that address the hypotheses of the present report. Details on behavioral results and fMRI activity analyses of this data set have been reported previously (von Kriegstein et al. [5]). In short, these results have shown that normal participants as well as prosopagnosics recognize speech from face-learned speakers significantly better than from occupation-learned speakers. This behavioral benefit correlated positively with the amount of BOLD activity in face–movement sensitive pSTS. For the voice-recognition task, only normal participants showed a significant performance benefit for face-learned speakers in contrast to occupation-learned speakers. This performance benefit correlated positively with the amount of BOLD activity level in face-identity sensitive FFA. For details on results and statistical tests used see von Kriegstein et al. [5].

### Functional Connectivity between Face-movement and Speech-intelligibility Areas (Analysis 1)

**Figure 3:**
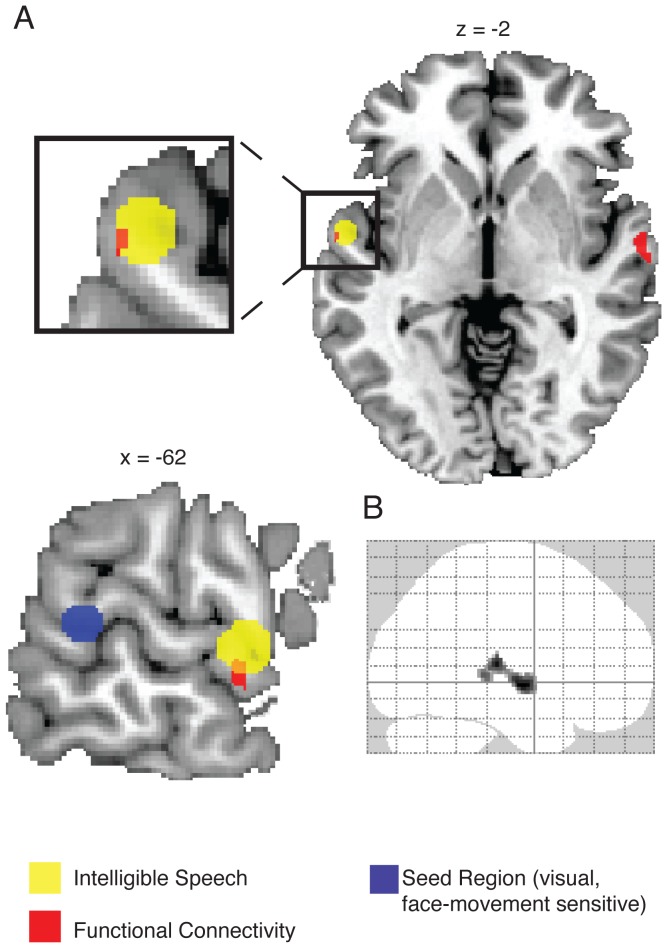
Functional connectivity of the visual face-movement sensitive STS (seed) during auditory speech recognition. (**A**) Increased functional connectivity (red) of the visual pSTS (blue) during recognition of speech from visually-known speakers (i.e., interaction learning type × task: (speech task/voice-face>speech task/voice-occupation)>(voice task/voice-face>voice task/voice-occupation)) is shown on an axial and sagittal brain slice. The auditory speech region of interest (ROI) in the anterior left STG/S is represented by a yellow sphere. The ROI was taken from a published coordinate [14]. On the sagital slice, the blue sphere schematically illustrates the posterior STS region in which individual seed regions were located. (**B**) Whole brain functional connectivity results are displayed in a “glass brain” at an uncorrected threshold of *p*<0.01, uncorrected, to show the specificity of the functional connectivity targets in the anterior STG/S.

The main hypothesis of the current study was that the face-movement sensitive pSTS and the speech-intelligibility sensitive aSTG/S would be functionally connected when recognizing auditory-only speech from visually-known (i.e., face-learned) speakers (Fig. 1). To address this hypothesis we performed a functional connectivity analysis for the psychological variable ((speech task/voice-face>speech task/voice-occupation)>(voice task/voice-face>voice task/voice-occupation)) using the face-movement sensitive pSTS as a seed region. This interaction specifically tests whether the functional connectivity between these two regions increases when participants recognize speech (compared to voices) of speakers that were previously learned together with their face (compared to an occupation symbol). As hypothesized, there was significant functional connectivity between face-movement sensitive pSTS and left aSTG/S in both groups (*p*<0.05 FWE corrected for aSTG/S) (Fig. 3A, Table 1). At a non-significant threshold of p<0.005 uncorrected, only three other clusters were found (Table 2). These were all located within the STS (Fig. 3). No other cortical areas showed increased functional connectivity, even when the statistical threshold was lowered to *p* 0.01 uncorrected whole-brain. This indicates that the connectivity to aSTG/S is very specific. There were no statistically significant differences between normal participants and developmental prosopagnosics in the functional connectivity to aSTG/S, even at a statistical threshold of *p = *0.05 uncorrected for the ROI. For completeness, we report differences between the two groups at the whole brain level at the non-significant threshold of *p*<0.001 uncorrected in Table S3.

**Table 1:** Auditory speech regions in aSTS.

			*MNI coordinates*			
*Study*	*Method*	*response sensitivity*	*x*	*y*	*z*	
Friederici et al. (2010)	fMRI	intelligible speech	−58	−4	4	*
Scott et al. (2000)	PET	intelligible speech	−66	−12	−12	n.s.
Obleser et al. (2007)	fMRI	intelligible speech	−57	−6	−3	*
Rosen et al. (2011)	PET	intelligible speech only	−52	8	−20	n.s.
		speech-typical amplitude and spectral modulations	−58	−2	−6	*
de Witt et al. (2011)	Meta-analysis	phrase-length speech recognition	−56	−8	−8	*

Published peak coordinates from a selection of studies investigating speech intelligibility/comprehension. Peak coordinates that yielded significant functional connectivity with the pSTS when used as a spherical volume of interest (r = 8 mm) are marked by an asterisk (* = p<0.05 FWE corrected). n.s. =  non-significant.

**Table 2:** Whole brain results for the functional connectivity with the pSTS.

Site	MNI peak coordinate			Z
*Both Groups*	*x*	*y*	*z*	
Left STS	−62	−6	−2	2.82*
Left STS	−66	−24	8	3.02
Right STS	62	−4	−6	2.77
Right STS	56	−32	2	2.71

For completeness, peak coordinates for the interaction contrast ((speech task/voice-face>speech task/voice-occupation)>(speaker task/voice-face>speaker task/voice-occupation)) are reported at a non-significant threshold of p<0.005, uncorrected.

* significant when corrected for the speech-intelligibility sensitive aSTS (p<0.05, FWE corrected).

For the statistical analysis, the ROI in aSTG/S was determined by a previously published coordinate from Friederici et al. 2010 (see methods). In a confirmatory analysis, we additionally used a wider selection of published coordinates found in speech intelligibility studies (Table 1). We found that the aSTG/S cluster showing increased functional connectivity with pSTS was co-localized with aSTG/S regions found in 4 of the 5 reported studies (*p*<0.05 FWE corrected for ROIs; see Table 1). These results remained significant (p<0.05 FWE corrected) after the exclusion of all participants for whom the visual face-movement sensitive STS was defined by the group average. To investigate whether connectivity between face-movement sensitive pSTS and speech-intelligibility sensitive aSTG/S may be mediated via a heteromodal region, we additionally tested for significant functional connectivity between the face-movement sensitive pSTS and potentially mediating heteromodal regions (pSTS, IFG, IPL). The regions were defined based on previous studies investigating integration of audiovisual speech (see Table 3). There was no indication of functional connectivity to either of these regions at the significance threshold (*p*<0.05 FWE-corrected for ROIs). None of the regions contained any peak voxels even at an uncorrected threshold (*p*<0.01 uncorrected within ROIs; see Table 3) and only two out of the 18 tested regions showed a connectivity peak at a threshold of p<0.05, uncorrected within ROIs.

**Table 3:** Multisensory interaction sites.

			*MNI coordinates*			
*Study (pSTS)*		*response sensitivity*	*x*	*y*	*z*	
Calvert et al. (2001)	Left	Superadditivity	−52	−38	8	n.s.
Calvert et al. (2000)	Left	Superadditivity x Congruency	−54	−50	7	n.s.
	Right		48	−58	19	n.s.
Miller & D’Esposito (2005)	Left	Audiovisual>silent baseline	−58	−40	12	n.s.
		Perceptual fusion>no fusion	−54	−28	−2	n.s.
Nath et al. (2010)	Left	Visual ∧ Auditory	−50	−47	14	n.s.
***Study (IFG)***		***response sensitivity***	***x***	***y***	***z***	
Skipper et al. (2005)	Left	Audiovisual>Audio-only	−46	21	5	n.s.
	Right		27	28	−10	n.s.
	Right		53	24	19	n.s.
	Right		48	12	21	n.s
	Left	Audiovisual>Visual-only	−47	21	16	n.s
	Right		52	23	15	n.s.
	Right		34	32	−4	n.s.
Ojanen et al. (2005)	Left	Incongruent>Congruent	−46	24	19	n.s.
			−45	18	8	n.s.
Miller & D’Esposito (2005)	Left	Audiovisual>silent baseline	−52	6	8	n.s.
***Study (IPL)***		***response sensitivity***	***x***	***y***	***z***	
Calvert et al. (2000)	Right	Superadditivity x Congruency	50	−35	44	n.s.
Skipper et al. (2005)	Right	Audiovisual>Visual-only	61	−46	21	n.s.

Published peak coordinates in a selection of studies investigating the posterior STS (pSTS), or the inferior frontal gyrus (IFG) and inferior parietal lobule (IPL) as a multisensory interaction site during speech perception. Peak coordinates were used as a spherical volume of interest (r = 8 mm). None of these coordinates were significantly functionally connected to the visual face-movement sensitive pSTS. If applicable, coordinates were transformed to MNI space. n.s. =  non-significant.

### Functional Connectivity between Face-identity and Voice-sensitive Areas (Analysis 2)

**Figure 4:**
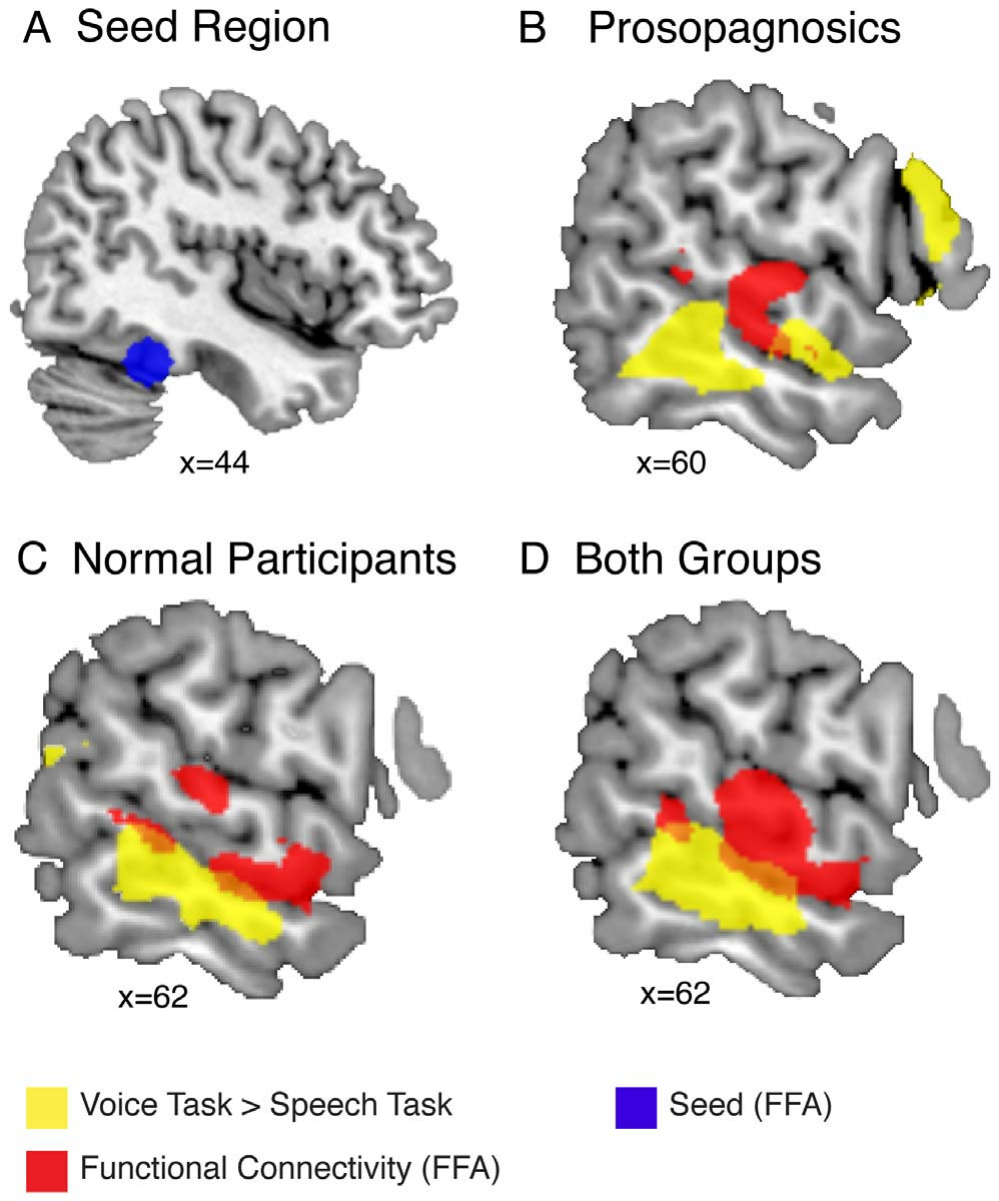
Functional connectivity of the FFA (seed) and the voice-sensitive areas in the right STS. (**A**) On the coronal slice, the blue sphere schematically illustrates the FFA for the group average. FFA seed regions were determined for individual participants. (**B**) Overlay of functional connectivity (PPI) and voice-sensitive areas is shown for prosopagnosics, normal participants (**C**) and both groups together (**D**). Clusters with increased functional connectivity (learning type x task: (voice task/voice-face>voice task/voice-occupation)>(speech task/voice-face>speech task/voice-occupation)) to the FFA are shown in red. Voice-sensitive areas identified by the task-related activity contrast (voice task>speech task) are shown in yellow.

To address the hypothesis that developmental prosopagnosics show a normal functional connectivity pattern between the FFA and the voice-sensitive regions in the right STS, we performed a functional connectivity analysis with the psychological variable ((voice task/voice-face>voice task/voice-occupation)>(speech task/voice-face>speech task/voice-occupation)) using the FFA as a seed region (Fig. 4A). This interaction specifically tested whether functional connectivity was increased when participants recognized voices (compared to speech) that were previously learned together with their face (compared to an occupation symbol). As hypothesized, in developmental prosopagnosics there was a significant functional connectivity between the FFA and a voice-sensitive right superior temporal area (*p*<0.042 FWE corrected for ROI; Fig. 4B). As already reported by several previous studies [8], [13], we also observed a similar functional connectivity between face- and voice sensitive areas for normal participants, although the results were only marginally significant (*p* = 0.055 FWE corrected for ROI; Fig. 4C). However, there were no significant group differences, even at an uncorrected statistical threshold, within voice-sensitive regions (*p*<0.01, uncorrected) or other brain regions (*p*<0.001, uncorrected, *k* = 8) indicating that the task-specific functional connectivity between face- and voice-sensitive areas is similar in both groups. This was supported by an analysis including both groups (developmental prosopagnosics and normal participants) that showed a significant functional connectivity between the FFA and the voice-sensitive right STS (*p*<0.027 FWE corrected for ROI; Fig. 4D). Whole brain results for this PPI at *p*<0.001, uncorrected, are displayed for information purposes in Table 4.

**Table 4:** Whole-brain results for functional connectivity analyses of FFA.

Site	MNI peak coordinate			*Z*	*P*
*Prosopagnosics*	*x*	*y*	*z*		
Right STS voice area	60	−8	−10	2.68	0.042 (FWE)
Right STS	58	−22	4	3.84	0.001
Left STS	−60	−30	2	3.86	0.001
*Normal Participants*					
Right STS voice area	64	−32	−4	2.03	0.055 (FWE)
Cerebellum	−48	−52	−34	3.69	0.001
*Both Groups*					
Right STS voice area	64	−16	−10	3.08	0.027 (FWE)
Left STS	−64	−12	−2	3.69	0.001

PPI peak coordinates in MNI space for the interaction contrast: ((voice task/voice-face>voice task/voice-occupation)>(speech task/voice-face>speech task/voice-occupation)).

Note that connectivity was statistically assessed based on the group-specific voice-sensitive region. As the location of voice-sensitive regions within the right STS are typically highly variable across subjects [37], we also expected the functional connectivity regions to be variable with respect to their absolute coordinate. Importantly, however, the regions in the right STS that were functionally connected to the FFA, showed a consistent overlap with the group-specific voice-sensitive regions. This overlap was significant in the region of interest analysis, but visual inspection of Figures 4B and 4C suggests that the overlap is only partial. This suggests that only parts of the voice-sensitive regions are functionally connected to the FFA.

## Discussion

The main finding of this study is that visual face-movement sensitive areas in the left pSTS communicate with an auditory speech-intelligibility sensitive area in the left aSTG/S during auditory-only speech recognition. In addition, we showed that people with a face-recognition deficit (developmental prosopagnosia) have a normal functional connectivity pattern between face-sensitive and auditory areas. This was the case for the functional connection between the visual face-movement sensitive pSTS and the auditory speech-intelligibility sensitive area in the left aSTG/S during the speech task, as well as between the face-sensitive FFA and the right temporal voice areas for the voice-recognition task.

The findings confirm and extend a recent audiovisual model of communication that assumes that visual face-sensitive brain areas are involved in the recognition of auditory-only communication sounds [5]. The core idea of this model is that the sensory analysis of auditory communication signals, such as speech and voice identity, is not exclusively accomplished by unisensory, auditory cortices as has been traditionally assumed [38], [39]; rather, it involves the contribution of visual, face-sensitive brain regions. The model was developed based on fMRI results that showed an activation of face-sensitive regions during auditory-only speech and voice recognition [5], [8], [13]. These activations were task-specific; the identity-sensitive FFA responded strongly during voice recognition, and the face-movement sensitive areas in the pSTS responded strongly during speech recognition. Moreover, the activity level in face-sensitive regions correlated with a behavioral recognition benefit, which suggests that face-sensitive regions contribute to auditory sensory processing. By “simulating” the face of a speaker, face-sensitive regions may feed the auditory system with predictions about the auditory speech signal and, thereby, optimize auditory recognition. Within this predictive-coding framework, functional interactions between the task-relevant sensory regions in the auditory and visual modality are a crucial pre-requisite. So far, evidence for such functional interactions has only been shown for voice recognition, with the FFA and the voice-sensitive right middle/anterior STS showing increased functional connectivity [8], [13]. The present study corroborates a central component of this model; it shows that the functional interaction between visual and auditory regions is a general mechanism during auditory-only communication that is also at work during speech recognition. Specifically, the current study suggests that the integration of visual information about face movements into auditory speech processing happens, at least partially, via functional connections between face-movement sensitive and auditory speech-intelligibility sensitive brain areas. The contribution of other regions cannot be entirely excluded, as the present analysis was focused on the connectivity profile of the face-movement sensitive pSTS. It is however worth noting, that no other, non-hypothesized brain region, which would motivate further analyses, has been found sensitive to speech recognition from face-learned voices in contrast to occupation-learned voices (p<0.05, FWE-corrected) von Kriegstein et al. [5]. Another limitation of the present analysis is that the employed method (PPI) cannot entirely assess the nature of this functional connection (i.e. whether it is mediated via a third region or directly). There are, however, two observations that rather speak for a direct connection. First, the regions interacting with the face-movement sensitive pSTS were confined to the bilateral STG/S; there were no other regions that showed a significantly increased connectivity. Second, a closer examination of typical multisensory interaction sites (i.e. potential mediating regions) did not reveal any connectivity to the face-movement sensitive pSTS, even at a lenient statistical threshold. We therefore speculate that there is a direct connection between the visual face-movement area and the auditory speech-intelligibility sensitive areas in the anterior temporal lobe. Such an interpretation would also be in line with anatomical findings. Although there have been no specific tracking studies of the connection between these two functionally defined areas, potentially corresponding anatomical regions are directly connected via the middle longitudinal fasciculus [40]–[42].

With regard to the functional specificity of the pSTS, it is difficult to entirely dissociate face-movement sensitive and multisensory regions in the STS as they are partially overlapping [43], [44]. In the current study, we are confident that the pSTS seed regions are primarily sensitive to face-movement, because of two reasons. First, we identified pSTS seed regions with contrasting BOLD responses to face-movements against a matched control condition (see methods) and at an individual level. Second, if the multisensory STS was important for auditory-only communication with visually-known speakers, it would presumably be similarly engaged during voice and speech recognition. However, this is neither the case in the connectivity analyses in the present study nor in the activity analyses (von Kriegstein et al., 2008). The anterior temporal lobe region that has been functionally connected to the visual face-movement sensitive area is part of the predominantly left-hemispheric anterolateral stream in the temporal cortex that is involved in speech perception [16], [19], [24]. Extending from mid STG to anterior STG/S regions, this stream has been proposed to reflect–in the anterior direction–increasing invariance to low-level acoustic features [17], [19], [24]. First evidence for such a directed stream comes from an effective connectivity study showing that intelligible speech is associated with an enhanced forward connectivity from posterior to anterior STS [45]. The anterior part of the STG/S has recently been highlighted as a crucial site for speech comprehension. Enhanced activity in the left aSTG/S is typically found when comparing intelligible speech with unintelligible speech-like sounds [14]–[16], and its activity is a reliable indicator of successful speech comprehension [19], [46]. Its causal role in auditory speech comprehension has recently been underlined by an electric stimulation study in which a transient lesion of the aSTS in an epileptic patient resulted in impaired sentence comprehension while leaving other auditory perception tasks (such as perceiving music or non-linguistic auditory sounds), as well as visual sentence comprehension, intact [20]. The exact role of this region is currently under debate. Rosen et al. [17] tried to disentangle acoustic and linguistic contributions to speech-intelligibility and showed that more posterior areas of the speech-intelligibility brain region are sensitive to the speech-typical acoustic complexity of sounds (i.e., frequency and amplitude modulations), and a more anterior region is largely independent of the acoustic form and is more sensitive to comprehension on a linguistic level. The aSTG/S region found in the present study is closest to a region that is sensitive to the speech-typical combination of temporal amplitude modulations and spectral content and insensitive to the linguistic content. We tentatively interpret this as evidence that the face-movement sensitive pSTS is functionally connected to a high-level auditory–sensory region, rather than an amodal region dealing with the processing of linguistic content. The functional connectivity of the pSTS to such an auditory area makes sense in the light of the current experimental design and within the framework of an audiovisual model of communication [5]: during the audiovisual training, participants were exposed to the audiovisual dependencies of the speaker’s moving face. It is, for example, known that the mouth opening of a speaker correlates with temporal amplitude modulations of the produced speech sounds, as well as the spectral content [47]. It is, therefore, conceivable that the face-movement sensitive pSTS and the speech-intelligibility sensitive area in aSTG/S are co-modulated during the audiovisual training, leading to a learning of speaker-specific regularities between visual and auditory speech. During the later test session, when the visual input is missing, these learned regularities allow for a visual “simulation” of the speaker that, in turn, helps to predict amplitude and spectral dynamics of the auditory signal.

In the current study, we used previously published coordinates representing speech-intelligibility sensitive regions in the left anterior STG/S. Ideally, the speech-intelligibility sensitive aSTG/S would have been determined within our own cohort of participants. Unfortunately, the scope of the current study did not allow for the additional integration of a speech-intelligibility design. In the second analysis (i.e. voice recognition), voice-sensitive regions were identified within our own cohort of participants. This approach has the advantage that it accounts for group differences and that we can be certain that the examined regions are indeed voice-sensitive. However, we are confident that the integration of published coordinates (that have been confirmed and replicated several times) into our analysis is a valid scientific approach (see, e.g. [48]–[51]) that may have its own merits, such as their full independence of the data to be analyzed. A correlation between the aSTG/S and speech recognition performance would have been a clear indication for the relevance of the aSTG/S during speech recognition in the present group of participants. However, we did not find such a correlation in the current data set, probably because the speech-recognition task was extremely easy for participants and consequently, the behavioral results are at ceiling with very little variance across participants. In the second analysis (i.e. voice recognition), voice-sensitive regions were identified within our own cohort of participants. This approach has the advantage that it accounts for group differences and that we can be certain that the examined regions are indeed voice-sensitive. This is especially important, because the location of voice-sensitive regions is variable over subjects [37].

Several studies have addressed the functional connectivity between auditory and visual regions in multisensory settings, i.e., when both visual and auditory speech stimuli are presented together or in close proximity. Exposing monkeys to dynamic audiovisual vocalizations induces functional interactions between the face-sensitive pSTS and a primary auditory region [11]. Also, in humans, relevant functional interactions during audiovisual speech have been shown to exist between visual, motion-sensitive areas and auditory regions surrounding Heschl’s gyrus [10], as well as between the multisensory pSTS and primary auditory and visual cortices [31]. One major difference between these previous studies and the present study is the use of audiovisual rather than auditory-only stimulus material. The concurrent stimulation of visual and auditory structures induces, most likely, strong bottom-up driven functional interaction within and between various levels of the sensory processing streams that are not necessarily expected under auditory-only conditions. Another crucial difference is that none of the above-mentioned studies aimed to investigate high-level task effects. The comparison of two different tasks (i.e., speech and voice recognition), rather than the comparison of different stimulation conditions, controls strictly for lower-level sensory processes and is therefore more likely to reveal connectivity differences between higher-level sensory areas that show preferential activity for different tasks.

For auditory-only speech perception, the present results suggest that the integration of visual and auditory information is, at least partially, based on the functional connection between relatively high-level, visual face-movement sensitive and auditory speech-intelligibility sensitive areas. The functional connectivity is specific to the speech task, suggesting that the interaction of the pSTS and the aSTG/S is not simply driven by the auditory-visual association inherent in the stimulus. Rather, the integration of facial information into the auditory speech analysis happens when speech comprehension is behaviorally relevant and when visual information can help to make perception more robust. This finding is in line with previous studies showing a functional connection between two other high-level sensory areas, i.e., face-sensitive FFA and voice-sensitive STS during auditory-only voice recognition that was also task-specific, i.e., increased for the voice compared to speech task [8], [13]. We, therefore, speculate that the neural processing of auditory communication in general, relies on the cross-modal interaction of task-relevant visual and auditory areas.

Functional connectivity was the same for developmental prosopagnosics and normal participants, indicating that the interaction of face-sensitive and auditory regions during both speech and voice recognition did not depend on the ability to recognize face identity. For speech recognition this was expected, because developmental prosopagnosics do not seem to have difficulties with face-movement perception in general, and they also use the face-movement sensitive area in the left pSTS to optimize auditory-speech recognition [5], [52]–[55]. In the case of voice recognition, the present finding substantiates a previous case study reporting normal functional connectivity in a single prosopagnosic between the FFA and voice-sensitive cortices during voice recognition [56]. The findings of the present study demonstrate that, for the relatively large sample size of 15 developmental prosopagnosics, the connectivity between the FFA and the voice-sensitive STS during voice recognition is preserved. Although developmental prosopagnosics have grey matter deficits in a fusiform region that might correspond to the FFA [57], most functional studies, in particular with larger sample sizes, have shown no consistent differences in the FFA activity level between developmental prosopagnosics and normal participants [5], [58]–[60]. Developmental prosopagnosics also show normal overall FFA activity levels during voice recognition. Unlike normal participants, however, their FFA activity levels do not correlate with the degree to which they use facial information during voice recognition [5]. This suggests that developmental prosopagnosics do not use the FFA to optimize voice recognition and that it is the integrity of the neural processing within the FFA that is compromised, rather than its overall activity level. The preservation of FFA functional connectivity in prosopagnosics during voice-recognition is in agreement with these findings. Importantly, this finding has implications for classical person-identity models [39]. Classical models suggest that identity information from faces and voices are integrated by supramodal areas dealing with person identity–the person identity node (PIN)–and do not foresee direct connections between face- and voice-sensitive areas. Following this account, the functional connectivity between face- and voice-sensitive areas found in normal participants would be mediated by the PIN rather than reflecting a direct integration mechanism. Because prosopagnosics do not recognize identity from faces, it is unlikely that a supramodal area mediates the FFA activity, i.e., that the voice-sensitive regions evoke a supramodal representation of the person, which in turn activates the FFA, because of face imagery or retrieval of the identity of the face. The preservation of functional connectivity in developmental prosopagnosics rather indicates that face- and voice-sensitive areas interact directly on a sensory level and that the FFA is activated independently of an accessible facial identity representation.

In summary, the current results suggest that functional connectivity between face-sensitive and auditory speech- and voice-sensitive regions represents a general mechanism by which learned visual speaker information is incorporated into the ongoing auditory speech and voice analysis, and that this integration is mainly implemented by task-specific functional connections between specialized visual face-sensitive and auditory speech/voice-sensitive brain regions.

## Supporting Information

Table S1 **Individual peak coordinates for visual pSTS that shows crossmodal activity when recognizing speech from familiar speakers.** Subjects are labeled n for normal subjects and p for prosopagnosics.
(DOCX)

Table S2 **Individual peak coordinates for the crossmodal FFA (i.e. responding to voices from familiar speakers).** Subjects are labeled n for normal subjects and p for prosopagnosics.
(DOCX)

Table S3 **PPI peak coordinate in MNI space for the interaction contrast: ((speech task/voice-face>speech task/voice-occupation)>(speaker task/voice-face>speaker task/voice-occupation)) for both groups.** The asterisk indicates that the p-value is FWE-corrected p-value for the region of interest. For completeness, group differences are also reported at an uncorrected threshold of p<0.001 and a cluster size of k = 8. STS - superior temporal sulcus.
(DOCX)
