# Supplementary material for: Functional Connectivity between Face-Movement and Speech-Intelligibility Areas during Auditory-Only Speech Perception
Source: PLoS One. 2014 Jan 23;9(1):e86325. doi: 10.1371/journal.pone.0086325 (PMC3900530; doi:10.1371/journal.pone.0086325)
Supplement: Table S1 — Individual peak coordinates for visual pSTS that shows crossmodal activity when recognizing speech from familiar speakers. Subjects are labeled n for normal subjects and p for prosopagnosics. (DOCX) [file pone.0086325.s001.docx]

**Table S1**:

|  | MNI coordinates | | |  |
| --- | --- | --- | --- | --- |
| Subjects | x | y | z | Z |
| n1 | -60 | -66 | 4 | 2.39 |
| n2 | -48 | -50 | 2 | 2.35 |
| n3 | -48 | -44 | 22 | 2.45 |
| n4 | -46 | -62 | 10 | 2.51 |
| n5 | -66 | -48 | 6 | 2.31 |
| n6 | -58 | -40 | 4 | 2.34 |
| n7 | -48 | -40 | 14 | 2.39 |
| n8 | -48 | -50 | 8 | 3.01 |
| n9 | - | - | - | - |
| n10 | - | - | - | - |
| n11 | -54 | -52 | 6 | 2.77 |
| n12 | -52 | -52 | 14 | 1.68 |
| n13 | -48 | -44 | -4 | 3.64 |
| n14 | -58 | -60 | 16 | 3.86 |
| n15 | -54 | -46 | 10 | 1.91 |
| n16 | -56 | -36 | 18 | 2.37 |
| n17 | -60 | -44 | 0 | 1.95 |
|  |  |  |  |  |
| p1 | - | - | - | - |
| p2 | -56 | -58 | -8 | 2.96 |
| p3 | - | - | - | - |
| p4 | -48 | -56 | -2 | 2.95 |
| p5 | -66 | -48 | 8 | 2.36 |
| p6 | -60 | -60 | 26 | 4.23 |
| p7 | -54 | -54 | 14 | 3.27 |
| p8 | -64 | -54 | 14 | 2.75 |
| p9 | -50 | -56 | 12 | 4.13 |
| p10 | -66 | -42 | 6 | 2.45 |
| p11 | -42 | -66 | 16 | 2.24 |
| p12 | -54 | -58 | -2 | 3.55 |
| p13 | -66 | -54 | 4 | 1.84 |
| p14 | -42 | -64 | 2 | 3.27 |
| p15 | -50 | -56 | -4 | 2.67 |
| p16 | -60 | -40 | 4 | 3.26 |
| p17 | -48 | -48 | 2 | 2.54 |
