# Supplementary material for: Functional Connectivity between Face-Movement and Speech-Intelligibility Areas during Auditory-Only Speech Perception
Source: PLoS One. 2014 Jan 23;9(1):e86325. doi: 10.1371/journal.pone.0086325 (PMC3900530; doi:10.1371/journal.pone.0086325)
Supplement: Table S2 — Individual peak coordinates for the crossmodal FFA (i.e. responding to voices from familiar speakers). Subjects are labeled n for normal subjects and p for prosopagnosics. (DOCX) [file pone.0086325.s002.docx]

**Table S2:**

|  | MNI coordinates | | |  |
| --- | --- | --- | --- | --- |
| Subjects | x | y | z | Z |
| n1 | 36 | -46 | -22 | 2.48 |
| n2 | 42 | -50 | -28 | 1.86 |
| n3 | 42 | -44 | -22 | 2.89 |
| n4 | 44 | -60 | -24 | 2.51 |
| n5 | 40 | -56 | -24 | 3.71 |
| n6 | 50 | -42 | -18 | 2.83 |
| n7 | - | - | - | - |
| n8 | 42 | -38 | -22 | 2.05 |
| n9 | 40 | -48 | -36 | 4.78 |
| n10 | 44 | -68 | -22 | 2.34 |
| n11 | 42 | -52 | -28 | 1.71 |
| n12 | 38 | -46 | -32 | 2.8 |
| n13 | 38 | -40 | -26 | 2.38 |
| n14 | - | - | - | - |
| n15 | 38 | -54 | -22 | 2.46 |
| n16 | 36 | -54 | -20 | 2.83 |
| n17 | 42 | -46 | -20 | 1.94 |
|  |  |  |  |  |
| p1 | 42 | -42 | -22 | 2.18 |
| p2 | 50 | -40 | -20 | 3.39 |
| p3 | 48 | -50 | -26 | 2.97 |
| p4 | 44 | -42 | -24 | 2.58 |
| p5 | 50 | -48 | -26 | 3.18 |
| p6 | - | - | - | - |
| p7 | 50 | -60 | -24 | 1.7 |
| p8 | 44 | -44 | -34 | 2.02 |
| p9 | 48 | -48 | -24 | 1.77 |
| p10 | - | - | - | - |
| p11 | 40 | -40 | -30 | 4.03 |
| p12 | 38 | -44 | -22 | 2.4 |
| p13 | 40 | -56 | -20 | 2.41 |
| p14 | 42 | -46 | -32 | 3.37 |
| p15 | 36 | -60 | -22 | 3.59 |
| p16 | 40 | -52 | -28 | 1.74 |
| p17 | 48 | -48 | -22 | 2.35 |
