# Supplementary material for: Functional Connectivity between Face-Movement and Speech-Intelligibility Areas during Auditory-Only Speech Perception
Source: PLoS One. 2014 Jan 23;9(1):e86325. doi: 10.1371/journal.pone.0086325 (PMC3900530; doi:10.1371/journal.pone.0086325)
Supplement: Table S3 — PPI peak coordinate in MNI space for the interaction contrast: ((speech task/voice-face>speech task/voice-occupation)>(speaker task/voice-face>speaker task/voice-occupation)) for both groups. The asterisk indicates that the p-value is FWE-corrected p-value for the region of interest. For completeness, group differences are also reported at an uncorrected threshold of p<0.001 and a cluster size of k = 8. STS - superior temporal sulcus. (DOCX) [file pone.0086325.s003.docx]

**Table S3:**

|  | Site | MNI peak coordinate | | | Z |
| --- | --- | --- | --- | --- | --- |
| *Both Groups* | | *x* | *y* | *z* |  |
|  | Left STS (anterior) | -62 | -6 | -2 | 2.82* |
| *Prosopagnosics > Controls* | |  |  |  |  |
|  | Right STS (anterior) | 64 | -8 | -14 | 3.38 |
|  | Parahippocampal  Gyrus | -34 | -20 | -18 | 3.34 |
